# Monitoring the quality of the water used in mobile dialysis services in intensive care units in the city of Rio de Janeiro

**DOI:** 10.1590/2175-8239-JBN-2020-0217

**Published:** 2021-07-23

**Authors:** Priscila Rodrigues de Jesus, Joana Angélica Barbosa Ferreira, Juliana dos Santos Carmo, Sheila Regina Gomes Albertino, Santos Alves Vicentini, Lisia Maria Gobbo dos Santos, Helena Pereira da Silva Zamith

**Affiliations:** 1 Fundação Oswaldo Cruz, Instituto Nacional de Controle de Qualidade em Saúde, Rio de Janeiro, RJ, Brasil.

**Keywords:** Dialysis Solutions, Microbiology, Renal Dialysis, Hemodialysis Solutions, Soluções para Diálise, Microbiologia, Dialise Renal, Soluções para Hemodialise

## Abstract

**Introduction:**

Monitoring water quality in mobile dialysis (MD) services, assessing critical points and characterizing the risks inherent in the process, is essential to avoid risks to the patient's health. This study evaluated the microbiological quality of water in the MD of 36 hospitals with intensive treatment in the city of Rio de Janeiro.

**Methods:**

204 water samples were collected from the points of entry to the network (NET), post-osmosis (PO) and dialysis solution (DS). The samples were evaluated for heterotrophic bacteria count, pathogen search, presence of endotoxins and aluminum content.

**Results:**

Bacterial contamination at 3 collection points in 36 hospitals was 30% (32/108); 42% from DS, 31% from PO and 17% from NET, with the presence of *Pseudomonas aeruginosa, Stenotrophomonas maltophilia , Burkholderia cepacia and Ralstonia pickettii* in the 3 points. Endotoxin concentrations above 0.25 EU/mL occurred in 77% of the samples (17/22) analyzed in the PO. In the aluminum content, values above 0.01 mg/L were presented in 47% (7/15) of PO samples and 27% (4/15) of NET samples. There is no specific legislation for water used in the MD; therefore, the limits of the RDC of the National Health Surveillance Agency (Anvisa) 11/2014 were used; which regulates conventional hemodialysis services.

**Conclusion:**

The results highlight the importance of evaluating water quality in MD services to ensure patient safety and support the sanitary monitoring of this process as a healthcare promoter.

## Resumo

**Introdução:**

Monitorar a qualidade da água nos serviços de diálise móvel (DM), avaliando os pontos críticos e caracterizando os riscos inerentes ao processo, é fundamental para evitar riscos à saúde do paciente. Este estudo avaliou a qualidade microbiológica da água na DM de 36 hospitais com tratamento intensivo no município do Rio de Janeiro.

**Métodos:**

Foram coletadas 204 amostras de água dos pontos de entrada da rede (REDE), pós-osmose (PO) e solução de diálise (SD). As amostras foram avaliadas quanto à contagem de bactérias heterotróficas, pesquisa de patógenos, presença de endotoxinas e teor de alumínio.

**Resultados:**

A contaminação bacteriana, em 3 pontos de coleta nos 36 hospitais, foi de 30% (32/108), sendo 42% provenientes da SD, 31% da PO e 17% da REDE, com presença de *Pseudomonas aeruginosa, Stenotrophomonas maltophilia, Burkholderia cepacia e Ralstonia pickettii* nos 3 pontos. Concentrações de endotoxina acima de 0,25 EU/mL ocorreram em 77% das amostras (17/22) analisadas na PO. No teor de alumínio, os valores acima de 0,01 mg/L foram apresentados em 47% (7/15) das amostras da PO e 27% (4/15) das amostras da REDE. Não existe uma legislação específica para água utilizada na DM; logo, foram utilizados os limites da RDC da Agência Nacional de Vigilância Sanitária (Anvisa) 11/2014, que regulamenta os serviços de hemodiálise convencional.

**Conclusão:**

Os resultados ressaltam a importância da avaliação da qualidade da água nos serviços de DM para garantir a segurança do paciente e subsidiar o monitoramento sanitário desse processo como um promotor de saúde.

## Introduction

Hemodialysis (HD) is an essential treatment for patients with chronic kidney disease (CKD) or acute renal failure (ARF); which occurs when the kidneys are unable to remove waste products from cellular metabolism or perform their regulatory functions^1^. In general, patients affected by AKI or in cases of CKD requiring hospitalization are submitted to mobile dialysis (MD), which occurs in the hospital setting^2^^,^ 3.

MD is a process performed on a HD machine connected to a portable osmosis device, which can be used in wards or intensive care units. The machine is connected to the water supply of the hospital network for the procedure to be carried out^4^.

During the process, the HD machine receives, through a vascular access, the patient's blood, which is driven by a pump to the dialyzer, where it is exposed to the dialysis solution (DS) in counter-parallel flow through a semi-permeable membrane, which removes excess fluid and toxins by diffusion and returns purified blood to the patient, in addition to restoring the blood acid-base and water and electrolyte balance^3^^,^ 5 ^,^ 6.

Water is essential in HD for diluting the polyelectrolytic concentrate for hemodialysis (PCHD) and obtaining the DS. In a conventional HD session, approximately 120 L of purified water is used, mixed in adequate proportions to the PCHD for blood clearance^7^.

The microbiological approach to water was considered when it was demonstrated that the high levels of Gram-negative bacteria in the DS were also responsible for pyrogenic reactions and cases of bacteremia in patients^8^, with species of the Pseudomonadales class being the most frequent, as they can grow in the circuits water and HD machines, and subsequently contaminate the DS. In addition, endotoxins from Gram-negative bacteria can penetrate the dialyzer's semipermeable membrane^7^^,^ 8 ^,^ 9.

Conventional HD services are regulated by the National Health Surveillance Agency (Anvisa) through the Resolution of the Collegiate Board (RDC) No. 11 of March 13, 2014, which provides for the Requirements of Good Operating Practices for Dialysis Services, and it deals with patient care, the structure necessary for the proper performance of the service, and defines the quality parameters to be met for treated water for HD.^10^

MD services do not have specific federal legislation that guides the monitoring of the quality of the service provided. Consequently, the parameters described in the Brazilian Pharmacopoeia for purified water can be used^11^. Anvisa's General Management of Technology and Health Services (GGTES) published Technical Note No. 006/2009, which aims to establish parameters for the execution of dialysis procedures in a hospital setting, outside dialysis services, and recommends the use of treated water in accordance with the drinking parameters established by the current legislation^12^.

The control of the chemical composition of the HD water is necessary due to complications associated with the intoxication of patients with calcium and magnesium, fluorine, chlorine, and aluminum compounds, among others present in the water, which can cause side effects such as nausea, vomiting and dizziness during the HD^13^^,^ 14 process.

The quality criteria related to the monitoring the treated water are related to the prevention of bacteremia and pyrogenic reactions. It is necessary to improve the monitoring of treated water for MD in order to learn about possible microbiological and chemical contamination, and to establish specific control strategies in relation to the contamination of the system^15^.

Many efforts have been made in Brazil in order to guide the quality of the MD service provided, until we can have legislation that regulates this service.

Studies to evaluate the quality of water in MD services are essential to support Anvisa's definition of specific parameters to control the quality of the water used, since a broad regulation applicable to the entire national territory would make the work more effective, being carried out by sanitary inspection agencies.

Thus, the objective of this study was to evaluate the quality of the water used in MD services in intensive care hospital units in the city of Rio de Janeiro.

## Methodology

In order to carry out this study, we visited hospital units to accompany inspections carried out by professionals from the State Sanitary Surveillance (VISA) of Rio de Janeiro and the National Institute for Quality Control in Health (INCQS), an integral part of the Oswaldo Cruz Foundation (Fiocruz), located in Rio de Janeiro. From these visits, it was possible to collect material to be analyzed in the laboratory. Participation in inspections and the collection of material were consented by all professionals involved in the process, and their identity and the location of the hospital units were preserved.

We collected 204 water samples in 36 hospital units located in the city of Rio de Janeiro from February 2017 to October 2018.

The maintenance of the portable osmosis and HD machines of the MD service performed in the hospital units was outsourced. An outsourced company cleaned the machines weekly, and the filtering membranes used were discarded. The MD machines were specific to each hospital unit, and they could be moved between wards and intensive care units (ICUs) within the unit.

During the MD process, the portable osmosis equipment was connected to a drinking water source in the hospital close to the patient, and the water, after being submitted to the reverse osmosis treatment, was directed to the machine to solubilize the PCHD and to carry out the HD process.

The samples for microbiological analysis came from three collection points: 1) water from the network entrance (hospital distribution water); 2) post-osmosis (water after treatment by portable reverse osmosis); and 3) dialysis solution (ready-to-use solution). For aluminum quantification, the samples were collected only at points 1 and 2.

### Specimen collection

We collected samples during the HD session into sterile glass bottles. We depyrogenized the vials for the endotoxin samples. We collected samples in conical bottom tubes, previously prepared with the addition of 1% nitric acid for aluminum quantification.

The samples were transported in thermoboxes with controlled temperature, and analyzed on the same day of collection.

### Microbiological analysis, quantification of endotoxins and the aluminum content of the samples

To count the heterotrophic bacteria, we used a pour plate method, with a 48-h incubation at a temperature of 32.5ºC ± 2.5oC. For this study, we used coliforms and Escherichia coli, incubated for up to 48h at 43oC ± 1oC in Mac Conkey broth. The quantification of endotoxins was performed by in vitro testing of Limulus amoebocyte lysate (LAL) using the gelation method. All the tests performed are described in the Brazilian Pharmacopoeia^11^.

The phenotypic identification of the microorganisms isolated in the water samples was carried out according to the methodology described by Jorgensen & Pfaller (2015)^9^.

We quantified the aluminum content using absorption spectrometry with a graphite oven, according to the methodology described by the American Public Health Association (APHA)^16^.

### Analysis of results

The results of the analyzes performed were interpreted according to the parameters and limits recommended by RDC 11/2014 for the post-osmosis and DS points, absence of Escherichia coli in 100 mL, heterotrophic bacteria count in a maximum of 100 colony-forming units (CFU)/mL (post-osmosis) and 200 CFU/mL in the DS and endotoxin concentration of up to 0.25 units of endotoxin (EU)/mL (post-osmosis) and maximum aluminum content of 0.01 mg/L^10^.

For the inlet water, the limits used were: absence of Escherichia coli and total coliforms in 100 mL, heterotrophic bacteria count 500 CFU/mL and maximum aluminum content of 0.2 mg/L according to Consolidation Ordinance nº 5, of September 28, 2017^17^.

## Results

We monitored 36 hospital units, 39% public and 61% private.

In microbiological tests, of the 108 samples collected at the three points (network entrance, post-osmosis and DS), 30% had a heterotrophic bacteria count above the limits recommended by RDC 11/2014^10^. The search for total coliforms and Escherichia coli did not show positive results. Table 1 shows the counts of heterotrophic bacteria found in the water and the DS samples, as well as the samples with negative results.

**Table 1:** Count of heterotrophic bacteria in water samples obtained from 3 different collection points in 36 hospital units in the city of Rio de Janeiro in mobile dialysis services in 2017 and 2018. results expressed in number of colony-forming units (cfu)/ml

Hospital Unit	Network inlet	Post-osmosis	Dialysis solution
**1**	<10	<10	**1.5 x 10^3^**
**2**	<10	<10	**1.0 x 10^3^**
**3**	<10	<10	<10
**4**	**1.9 x 10^3^**	**2.3 x 10^4^**	**2.0 x 10^3^**
**5**	<10	**1.0 x 10^3^**	**1.2 x 10^3^**
**6**	<10	<10	<10
**7**	**3.6 x 10^2^**	**1.5 x 10^3^**	**1.0 x 10³**
**8**	<10	**1.0 x 10^3^**	**1.2 x 10^4^**
**9**	<10	<10	<10
**10**	<10	<10	<10
**11**	<10	<10	<10
**12**	<10	**1.1 x 10^3^**	**2.5 x 10^4^**
**13**	<10	<10	<10
**14**	<10	<10	<10
**15**	<10	<10	<10
**16**	<10	<10	<10
**17**	<10	<10	**2.0 x 10^4^**
**18**	<10	<10	<10
**19**	<10	<10	<10
**20**	<10	<10	<10
**21**	**1.9 x 10^2^**	**2.3 x 10^4^**	**2.0 x 10^3^**
**22**	<10	<10	<10
**23**	**2.6 x 10^3^**	**1.2 x 10** ^3^	**1.6 x 10^3^**
**24**	**1.5 x 10^4^**	**2.1 x 10^3^**	**2.5 x 10^3^**
**25**	<10	<10	<10
**26**	<10	<10	<10
**27**	**1.6 x 10^4^**	**2.1 x 10^4^**	**3.0 x 10^4^**
**28**	<10	<10	<10
**29**	<10	<10	<10
**30**	**1.0 x 10^4^**	**1.2 x 10^4^**	**1.4 x 10^4^**
**31**	<10	<10	<10
**32**	3.3 x 10^2^	**1.0 x 10^2^**	**1.5 x 10^3^**
**33**	<10	<10	<10
**34**	<10	<10	<10
**35**	<10	<10	<10
**36**	**1.0 x 10^3^**	**1.2 x 10^4^**	**1.4 x 10^4^**

The bacterial count values above the limits recommended by RDC 11/2014 of 500 UFC/mL for incoming network samples, 100 UFC/mL for post-osmosis samples and 200 UFC/mL for dialysis solution were highlighted in bold^10^^,^ 17.

**Graph 1:**
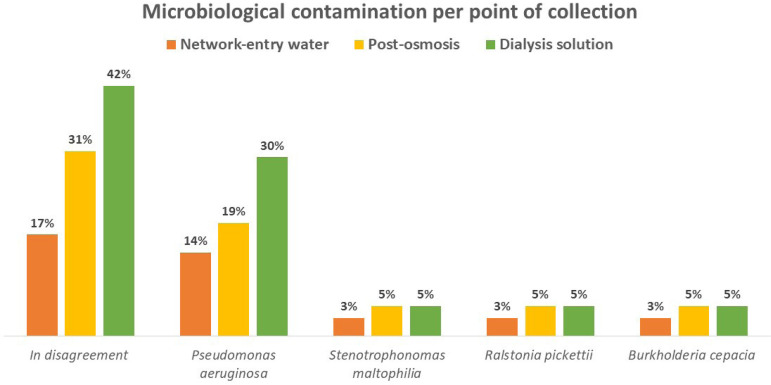
percentage of tap water samples, post-osmosis and dialysis solution with total number of aerobic bacteria in disagreement with the established by legislation (rdc 11/2014). samples collected in 36 hospitals in the city of rio de janeiro in mobile dialysis services in 2017 and 2018.

Graph 1 shows the percentage of water samples collected in 36 hospital units per collection point that presented microbial contamination above the limit recommended by legislation^10^^,^ 17, in mains water 6/36 (17%), 11/36 (31 %) in post-osmosis and 15/36 (42%) in DS. Furthermore, it shows the presence of microorganisms, *Pseudomonas aeruginosa, Stenotrophomonas maltophilia, Ralstonia pickettii and Burkholderia cepacia* isolated from the samples. The points of post-osmosis and DS were considered the most critical in the process, mainly regarding contamination by *Pseudomonas aeruginosa*.

Microbiological contamination per point of collection Network-entry water - Post-osmosis -

Regarding the quantification of endotoxins, 66 samples from 22 hospital units were analyzed (Table 2), and at the post-osmosis collection point, for which the limit value of 0.25 EU/mL^10^ is recommended, 77% presented values greater than 0.5 EU/mL.

**Table 2:** Quantification of endotoxins by the lal^1^ test - gelation method in water samples obtained from 3 different collection points in 36 hospital units in the city of Rio de Janeiro in mobile dialysis services in the years 2017 and 2018. results expressed in number of endotoxin units (eu)/ml

Hospital units	Pre-osmosis	Post-osmosis	Dialysis solution
1	> 0.5	**> 0.5**	> 0.5
2	> 0.5	**> 0.5**	> 0.5
3	< 0.125	< 0.125	> 0.5
4	> 0.5	**> 0.5**	> 0.5
5	> 0.5	**> 0.5**	> 0.5
6	> 0.5	**> 0.5**	> 0.5
7	> 0.5	**> 0.5**	> 0.5
8	≤ 0.25	**> 0.5**	> 0.5
9	> 0.5	**> 0.5**	> 0.5
10	< 0.125	< 0.125	< 0.125
11	> 0.5	**> 0.5**	> 0.5
12	< 0.125	**> 0.5**	> 0.5
13	> 0.5	**> 0.5**	> 0.5
14	< 0.125	< 0.125	< 0.125
15	> 0.5	**> 0.5**	> 0.5
16	> 0.5	**> 0.5**	> 0.5
17	< 0.125	< 0.125	> 0.5
18	< 0.125	< 0.125	< 0.125
19	> 0.5	**> 0.5**	> 0.5
20	> 0.5	**> 0.5**	> 0.5
21	> 0.5	**> 0.5**	> 0.5
22	≤ 0.25	**> 0.5**	> 0.5

1 Limulus amoebocyte lysate. Maximum limit of bacterial endotoxin for water treated for hemodialysis (collection point: post-osmosis) is 0.25 EU/mL10. Values above this limit are highlighted in bold.

Although the legislation does not recommend the quantification of endotoxins in the DS, we did this in our study, to allow comparison between the points.

In the aluminum quantification test, water samples were analyzed at points of entry into the network and post-osmosis collected from 22 hospital units in 2017 alone. Table 3 shows the results obtained in the aluminum analyzes from a total of 44 samples collected, of which 32% (14/44) could not be analyzed - which was considered a limitation. Of the remaining 30 samples, 37% (11/30) were found to be non-compliant, with aluminum values ​​above the limits recommended by the RDC 11/2014.

**Table 3:** Quantification of aluminum content in water samples obtained from the mains and from hemodialysis water collected in 22 hospital units in the city of rio de janeiro with mobile dialysis services in 2017. results expressed in aluminum concentration (mg/l)

Hospital units	Network entry	Post-osmosis
1	**> 0.27**	**> 0.27**
2	< 0.01	< 0.01
3	< 0.01	**> 0.27**
4	**> 0.27**	**> 0.27**
5	**0.3 ± 0.04**	**0.2 ± 0.04**
6	NR	NR
7	< 0.01	**> 0.27**
8	NR	NR
9	< 0.01	< 0.01
10	< 0.01	< 0.01
11	NR	NR
12	NR	NR
13	< 0.01	< 0.01
14	NR	NR
15	< 0.01	**> 0.27**
16	< 0.01	< 0.01
17	NR	NR
18	< 0.01	< 0.01
19	< 0.01	< 0.01
20	NR	NR
21	**> 0.27**	**> 0.27**
22	< 0.01	**> 0.27**

Aluminum limit value of 0.2 mg/L for mains water19 and 0.01 mg/L for post-osmosis^10^. Values above these limits highlighted in bold correspond to samples in disagreement with what is recommended by the RDC 11/2014.NP: analysis not performed.

The reduction of samples for quantification of endotoxins occurred due to the delay in the acquisition of kits for the LAL assay. In the case of aluminum quantification, the reduction was due to problems in the analysis equipment during the study period.

## Discussion

The absence of specific legislation for the monitoring of MD is a complicating factor, since the control that should be carried out periodically by the sanitary inspection bodies is not as efficient as in the conventional HD procedure, regulated by RDC nº 11 of 2014^10^. However, this RDC is not the best reference, since the treatment performed for water in MD services is different and less controlled than in conventional HD services.

Efforts have been made to guide MD services in the country; an example is the Health Department Resolution (SESA) No. 437/2013 of the Paraná state government, which provides for conditions for MD intra-hospital units outside the dialysis unit, through its own or outsourced services. However, this Resolution is only statewide and does not establish specific parameters for monitoring water quality^4^.

The results obtained made it possible to evaluate different aspects in relation to studies in this area, in which little is discussed about the structural issues of the services, such as critical points of the process and the proposal of other aspects to be analyzed and possible changes that could provide better results in the services. The study by Almeida and Batalha (2018)^18^, referring to an integrative literature review on the monitoring of dialysis services, highlighted as its main conclusion the need for studies that correlate the structure of services to the adequacy of processes and the evaluation through the results obtained in patients.

The HD water quality control is a public health problem on a worldwide scale, with quality standards being recommended in all countries. The European Renal Association recommends, for HD water, the limit of total count of heterotrophic bacteria of 100 CFU/mL, and 0.25 EU/mL for the quantification of bacterial endotoxin^19^. The Japanese Society for Dialysis Therapy recommends a count of heterotrophic bacteria below 100 CFU/mL, and a maximum of 0.05 EU / mL of endotoxin^20^. In the United States of America (USA), the maximum count limit for heterotrophic bacteria is 100 CFU/mL, and 0.25 EU/mL for endotoxin at all points except for the DS, for which the limit is 0,5 EU/mL^21^.

In the USA, the concern with the occurrence of bacteremia in patients undergoing the HD procedure has stimulated policies to improve processes with the use of ultrapure DS, with water treated by ultrasound and a maximum limit of 0.1 CFU/mL for counting heterotrophic bacteria. However, this type of water treatment is not yet mandatory and is still being studied^22^.

In 2014, MD services in the state of Rio de Janeiro were monitored by the CNPq/INCQS project, CNPq/Anvisa No. 05/2014 - Health Surveillance Research, through which 25 hospital units with MD were covered. The results of the study showed the need for continued monitoring of this service, since the number of aerobic bacteria, the presence of endotoxins and aluminum were high compared to those established by RDC No. 11 of 2014. Among the microorganisms surveyed, the species most often found was *Pseudomonas aeruginosa, followed by Escherichia coli, Stenotrophomonas maltophilia, Ralstonia pickettii and Burkholderia cepacia, and to a lesser extent Pseudomonas stutzeri, Acinetobacter anitratus, Acinetobacter baumannii, Acinetobacter calcoaceticos sp. lowffii, Sphingomonas paucimobilis, Brevundimonas diminuta, Moraxella osloensis, Moraxella lacunata, Moraxella henylpyruvia, Moraxella atlantae, Achronobacter xylosoxidans*, which are commonly found in water samples^23^.

Bacteria of the Pseudomonas^24^^,^ 25 and Sphingomonas^26^ genera are commonly found in water samples from treatment systems for HD. Special attention should be paid to *Pseudomonas aeruginosa* - an opportunistic pathogen that occurs in hospitalized patients, particularly in people with poor health. Its ability to form biofilms along the ducts of the system is an important specificity that allows recurrent contamination in pipes and equipment for hospital use with difficult control^26^.

The search *Pseudomonas aeruginosa* in the monitoring of water treated for HD is already recommended by the American Pharmacopoeia^27^ and the Brazilian Pharmacopoeia^11^ in ultra-pure waters. Thus, it would be important to include them in the regulations of the current Brazilian legislation, for the control of the quality of the water used in dialysis services, given its resistance to most antimicrobials and genetic diversity^28^.

Infections by P*seudomonas aeruginosa* often acquire a persistent character, and the strains can undergo a phenotypic change, acquiring adherence capacity, due to the formation of biofilms, which makes its eradication more difficult^26^^,^ 28.

Studies correlate the high concentration of endotoxins and the presence of bacteria in the DS with the occurrence of typical symptoms of pyrogenic reactions (endotoxemia) in patients^5^, which can be supported by evidence that, in high concentrations, bacterial endotoxins can cross the dialyzer membrane that presents minimal ruptures or even through intact membranes, determining symptoms in patients^29^.

The physiological activities of the lipopolysaccharide (LPS) are mediated mainly by lipid A, which causes a potent biological modifying response by stimulating the mammalian immune system. However, to induce a response on the organism, lipid A needs to be released in soluble form in vivo from cell lysis^30^^,^ 31.

In the present study, most clinics (77%), that is, 17 out of 22 hospital units had elevated values ​​of bacterial endotoxin in the LAL test. Microbiological tests often do not detect a bacterial count above the limit recommended by the legislation; however, in the quantification of endotoxins by the LAL test, as it is a test more sensitive to microbial degradation products, the presence of the microorganism can be perceived, even if it is not possible to quantify it^8^^,^ 30.

Endotoxins are frequent contaminants in aqueous/physiological solutions. Due to the various biological effects *in vivo* and *in vitro*, its detection and removal are essential to ensure patient safety during the HD^31^ procedure.

Another strategy for detecting contamination and/or quality deviations in the water used in MD is the use of molecular methodologies such as real-time polymerase chain reaction (PCR) or qPCR and metagenomics analyses - widely used in environmental water analysis is promising for treated water samples for HD, for enabling the analysis of viable non-cultivable microorganisms.^32^

Monitoring the presence of chemical elements in water for HD is important due to the risk of intoxication when their presence exceeds the concentration tolerated by the body, especially when it comes to patients undergoing HD. Aluminum is one of the most abundant elements in the earth's crust; however, it is not an essential element for the human body and its importance to health lies in the toxic and accumulative effect on the body^33^.

Of the sources of aluminum contamination, drinking water is one of the most significant, due to contact with the soil, and its concentration depends on the water pH. In addition, it is used in the treatment of drinking water, as a chelator, reducing the number of particles, to improve the appearance of the water^34^.

The aluminum action mechanism is not well understood, being considered a neurotoxic chemical agent, but for which there is little documented information regarding the molecular aspects of its cytotoxicity. Aluminum, when deposited at the junctions of calcified and non-calcified bones, becomes an obstacle to the incorporation of calcium by hydroxyapatite^33^^,^ 34.

The DS used in the HD process results from the dilution of PCHD with water treated by reverse osmosis. DS contamination can originate from both sources. However, the contamination of the water used in the treatment for HD by aluminum has always been pointed out as the main responsible for the encephalopathy, anemia and osteoarthritis seen in dialysis patients^35^.

Regarding the DS, the occurrence of contamination may be associated with contamination of the entire process, as the dialyzer used by these patients in MD is disposable. In addition, the non-sterile PCHD contribution, which is diluted in HD water in the proportion of 4:1 during the procedure, should be considered at this collection point, which may be another contamination factor; however, its handling must follow specific criteria, to avoid problems^12^.

The chlorine added to the distribution water makes the point of the network less likely to be contaminated, even if the collection is carried out in flasks prepared with chlorine-neutralizing solution, avoiding false negative results.^35^ The counts of heterotrophic bacteria above that recommended at the point of post-osmosis can result from the distribution water, from the portable osmosis device or even from the circuit through which the water circulates to the patient.

Conventional dialysis services have strict water treatment, unlike MD, in which there is no previous treatment of water to be used in reverse osmosis. Consequently, the MD contamination is a worrying factor that further arouses the need to monitor this service.

Despite reports of outbreaks associated with the quality of water treated for dialysis, cases are still very underreported, as only tragic events tend to be published, which does not allow us to assess the real frequency of adverse effects arising from dialysis treatment related to the treated water.

Ensuring and maintaining the quality of the water used in HD procedures is essential for the quality of life of patients with kidney problems, as the absence of efficient renal elimination poses risks to the lives of these patients.^35^

Additional studies addressing water monitoring, the occurrence of adverse events associated with contamination problems and the correlation between water contamination and the involvement of patients undergoing dialysis are necessary to support new directions of hemodialysis treatment offered in our country.

## Conclusion

The results obtained in this study underscore the importance of continuous and specific monitoring of the MD service and the need to develop a specific norm for its regulation, in order to subsidize the practices of the current inspection agencies and standardize the services offered to patients, in order to correct possible flaws in the process.

We expect that there will be encouragement for new studies in the area to assess the reality of the service in different regions of the country and to support the creation of legislation that meets the need and guides the existing and new MD services, contributing to the quality of life of acute renal patients.
